# Cerebrospinal Fluid Hypovolemia and Posterior Reversible Encephalopathy Syndrome

**DOI:** 10.3389/fneur.2020.00591

**Published:** 2020-06-23

**Authors:** Yuan-yuan Zheng, Xiong-peng Weng, Fang-wang Fu, Yun-gang Cao, Yan Li, Guo-qing Zheng, Wei Chen

**Affiliations:** ^1^Department of Neurology, The Second Affiliated Hospital and Yuying Children's Hospital of Wenzhou Medical University, Wenzhou, China; ^2^Department of Radiology, The Second Affiliated Hospital and Yuying Children's Hospital of Wenzhou Medical University, Wenzhou, China

**Keywords:** posterior reversible encephalopathy syndrome, cerebrospinal fluid hypovolemia, intracranial hypotension, dural puncture, epidural analgesia, cerebral hyperperfusion

## Abstract

Posterior reversible encephalopathy syndrome (PRES) is a reversible neuroradiological syndrome characterized by reversible vasogenic edema. The pathophysiological mechanism is still unclear, but PRES may be triggered by various etiologies. To date, only a few PRES cases linked to cerebrospinal fluid (CSF) hypovolemia were reported. The association between PRES and CSF hypovolemia needs to be explored. We presented a case of PRES with CSF hypovolemia as a result of an inadvertent dural puncture and reviewed the literature to identify the clinical characterization and pathophysiological mechanism of PRES following CSF hypovolemia. A total of 31 cases of PRES-CSF hypovolemia was included for analysis. The median age was 33 years, with a notable female predominance (87.1%). Fifteen patients (48.4%) didn't have either a history of hypertension nor an episode of hypertension. The most common cause of CSF hypovolemia was epidural or lumbar puncture (*n* = 21), followed by CSF shunt (*n* = 6). The median interval between the procedure leading to CSF hypovolemia and PRES was 4 days. Seizure, altered mental state, and headache were the most frequent presenting symptom. The parietooccipital pattern was most frequent (71.0%). Conservative management remains the mainstay of treatment with excellent outcomes. Three patients had a second episode of PRES. CSF hypovolemia is a plausible cause of PRES via a unique pathophysiologic mechanism including arterial hyperperfusion and venous dysfunction. Patients with CSF hypovolemia is more susceptible to PRES, which is potentially life-threatening. Given that CSF hypovolemia is a common complication of anesthetic, neurological, and neurosurgical procedures, PRES should be early considered for prompt diagnosis and appropriate management.

## Introduction

Posterior reversible encephalopathy syndrome (PRES), initially described by Hinchey et al. in 1996 (1), refers to a reversible clinical and neuroradiological syndrome characterized by acute headache, seizures, visual disturbances, impaired consciousness, focal neurological deficits, or combinations of them (2). The typical finding in neuroimaging is reversible vasogenic edema in subcortical white matter dominating in the bilateral posterior parieto-occipital region (2, 3). An increasing number of predisposing factors for PRES have been recognized including eclampsia, hypertensive crisis, organ transplantation, sepsis, subarachnoid hemorrhage (SAH), autoimmune disorders, renal insufficiency, and various immunosuppressive drugs (2, 4). The mechanism of PRES remains controversial. Hypertension/hyperperfusion theory and vasoconstriction/hypoperfusion theory have been commonly proposed to explain the pathophysiology of PRES (2, 5).

Cerebrospinal fluid (CSF) hypovolemia, which is used to be referred to as intracranial hypotension (IH) synonymously, is increasingly recognized as a critical but often a misdiagnosed cause of new-onset cephalalgia (6, 7). Usually, it included IH, but it was not an unequivocal definition of IH as a normal or even an increased CSF pressure was not rare in reported cases (8). It is usually triggered by dural puncture, lumbar puncture, spinal surgery, lumboperitoneal shunt, or other spontaneous reasons (6). Atypical clinical presentations including non-orthostatic headaches, visual defects, neurocognitive decline, epilepsy, and focal neurological deficits, which are similar to PRES, have already been reported. Recently, the association between PRES and CSF hypovolemia has started to emerge in the neurology (9–17), neurosurgery (18–24), and anesthesiology literature (10–13, 16, 19, 25–35). However, the association between PRES and CSF hypovolemia has not been fully elucidated.

To our knowledge, there was no systematic review exploring the pathogenesis, clinical and imaging characteristics, and management of PRES in patients with CSF hypovolemia. Herein, a case of PRES who suffered CSF hypovolemia after an inadvertent dural puncture was presented with potential evidence of hyperperfusion. Then, a systematic analysis of published literature was undertaken to reveal the possible association between PRES and CSF hypovolemia.

## Methods

The information of the patient from the department of Neurology of our hospital was collected for a preliminary analysis. The additional 30 cases (29 articles) in the PubMed and Web of Science database from inception to July 2019 using a combination with “PRES” and various terms related to CSF hypovolemia or high risks of CSF hypovolemia including “cerebrospinal fluid hypovolemia,” “intracranial hypotension,” “CSF leakage,” “epidural puncture,” “epidural anesthesia,” “spinal puncture,” “spinal anesthesia,” “lumbar puncture,” “cerebrospinal fluid shunt,” “spinal surgery,” and “cranial surgery.” A standardized form was applied to collect clinical information from each eligible article including demographic characteristics, related medical history, the probable cause of CSF hypovolemia, clinical manifestations, magnetic resonance (MR) findings (both PRES and CSF hypovolemia), treatment, and clinical outcome. The flow diagram was shown in the Supplementary Material.

Written informed consent for participation, data collection, and publication was obtained from the patient. Because this is a case report and review of literature, no research legal, and ethical approval is required.

### Case Presentation

A 30-year-old woman, gravida 3 para 0, without a previous history of hypertension, presented to the Department of Obstetrics at 40 weeks' gestation. Laboratory investigations at admission remained within the normal range. Epidural analgesia was planned for painless labor. An inadvertent dural puncture occurred in the first procedure. Then, no complication was found in the repeated epidural procedure. Her blood pressure remained consistently normal throughout labor, delivery, and the immediate postpartum period. Two hours after delivery, she complained of mild neck pain that resolved after receiving 2,000 ml Ringer's solution.

**Figure 1:**
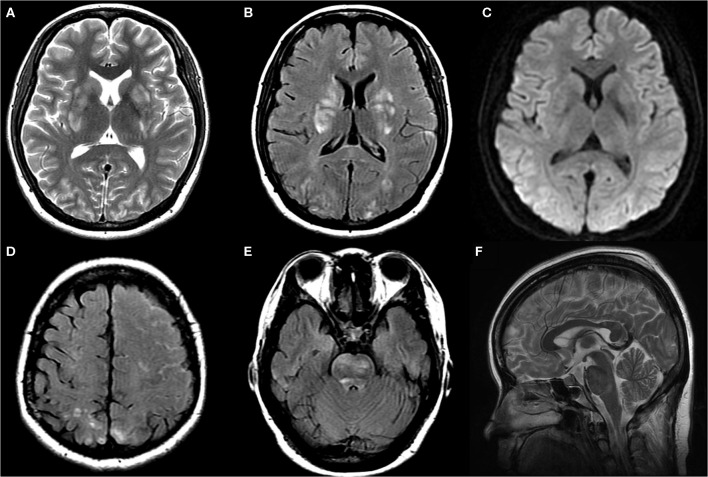
Axial T2WI **(A)**, axial FLAIR **(B,D,E)**, axial DWI **(C)** and sagittal T2WI **(F)** images at symptom onset: T2WI, FLAIR, and DWI images demonstrated hyperintensity without diffusion restriction in bilateral parieto-occipital region, basal ganglia, and brainstem. FLAIR images demonstrated left frontoparietal sulcus subarachnoid hemorrhage. Axial T2WI images demonstrated mild enlargement of pituitary and dural sinuses.

**Figure 2:**
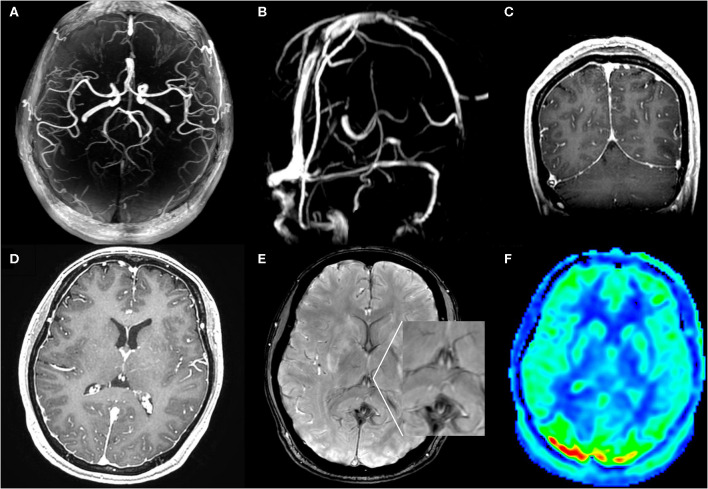
MR angiography **(A)**, MR venography **(B)**, post contrast T1WI **(C,D)**, SWI **(E)**, and ASL **(F)** MRI images at symptom onset: MR angiography and venography were negative for aneurysms, venous thrombosis, and cerebral vasospasm. SWI images showed lack of the normal hypointensity in deep venous system. Coronal and axial T1WI images with gadolinium-enhancement showed diffuse enhancement of the dura. ASL images showed hyperperfusion areas in bilateral occipitoparietal lobe.

**Figure 3:**
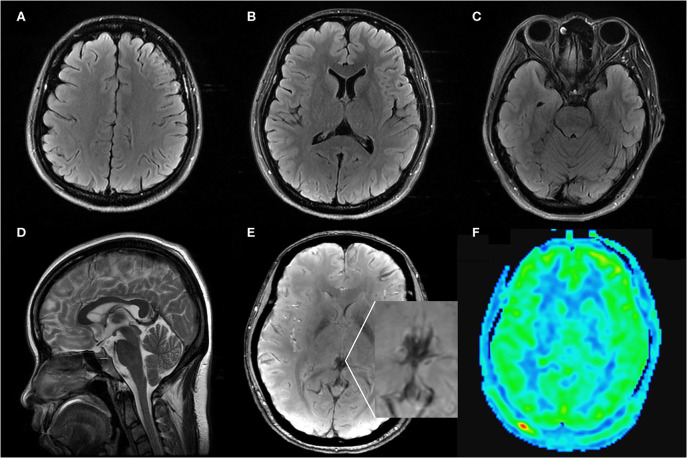
Axial FLAIR **(A–C)**, sagittal T2WI **(D)**, SWI **(E)**, ASL **(F)** MRI images on follow up: Axial FLAIR imaging demonstrated complete regression of vasogenic edema and convexity SAH. Sagittal T2WI images showed regression of the engorgement of the pituitary and dural sinuses. SWI and ASL images showed the normalization of the signal of the deep venous system and the CBF in bilateral occipitoparietal lobe.

On postpartum day 2, she developed a moderate postural occipital headache. In the absence of other focal neurological deficits, postdural puncture headache was diagnosed. The patient was managed with non-steroidal anti-inflammatory agents, hydration, and strictly bed rest. The epidural blood patch (EBP) was recommended as the following therapeutic measure, but the patient refused. On postpartum day 3, the patient complained of progressively worsening postural headache, nausea, and photophobia. The patient had to keep a recumbent posture to relief. The blood pressure was noted elevate to an average level of 140/85 mmHg and a highest-level of 178/96 mmHg. Nifedipine was taken to control hypertension. Then, the blood pressure was under 150/90 mmHg. On the early morning of postpartum day 4, the patient became confused when she waked up and turned to a supine position with a blood pressure of 131/90 mmHg. After a few minutes, she had a generalized tonic-clonic seizure which was controlled by diazepam. After she regained consciousness, she complained of diplopia and severe headache in occipital and left frontal region. Neurological examination revealed left abducens nerve palsy, right hemianesthesia, horizontal nystagmus, right tongue paralysis, and right Babinski sign. Diazepam and magnesium sulfate were taken with a concern that the patient was developing postpartum eclampsia. Six hours later, brain magnetic resonance imaging revealed vasogenic edema in the bilateral parieto-occipital regions, basal ganglia, and brainstem (Figures 1A–E). Convexity SAH was identified in the left frontal lobes (Figure 1C). MR angiography and venography were negative for aneurysms, venous thrombosis, and cerebral vasospasm (Figures 2A,B). The arterial spin labeling perfusion (ASL) imaging showed hyperperfusion areas in the bilateral occipitoparietal lobe (Figure 2F). On susceptibility-weighted imaging (SWI), the commonly marked hypointensity of the cerebral deep venous system was absent, suggestive of blood oxygen level dependent (BOLD) effect probably induced by cerebral hyperperfusion (Figure 2E). In addition, brain MR showed signs of intracranial hypotension including diffuse enhancement of the dura (Figures 2C,D), mild enlargement of pituitary and dural sinuses (Figure 1F), and slightly sagging of brainstem and cerebellum (Figure 1F). Thus, PRES and IH was the diagnosis. Over the following hours, the patient remained normal blood pressure and seizure-free. Magnesium sulfate infusion and diazepam were stopped. The patient was treated with intravascular rehydration which was used to prevent the progression of IH and SAH-induced cerebrovascular spasm. On postpartum day 14, the patient had a full recovery without any headache and neurological deficits. Follow-up MR imaging showed the complete disappearance of vasogenic edema, venous engorgement and convexity SAH (Figures 3A–D), together with the normalization of the signal of the deep venous system in SWI (Figure 3E) and the cerebral blood flow (CBF) in the bilateral occipitoparietal lobe (Figure 3F).

## Results

In total, we collected the data on 31 patients (30 patients from literature and our patient) for descriptive analysis. The detailed data of cases were summarized in Table 1.

**Table 1:** Characteristics and clinical manifestations of cases diagnosed with PRES and CSF hypovolemia.

**No**	**References**	**Age/Sex**	**Related history**	**Highest BP (mmHg)**	**Cause of CSF leak**	**Clinical manifestation**	**Time of PRES**	**PRES patterns**	**Edema grading**	**Atypical image of PRES**	**Treatment for IH**	**Edema resolution**	**Relapse**	**Outcome**
1	Moriarity et al. (18)	19/M	Hydrocephalus	200/130	Tumor resection, V-P shunt	Headache, altered mental status, GTCS, disturbed vision	2 h	Parieto-occipital	Severe	Cytotoxic edema	Conservative management	Incomplete	No	Mildly disconjugate gaze
2	Prout et al. (27)	32/F	Cesarean delivery	160/70	Spinal anesthesia	Headache, GTCS, disturbed vision	15 h	Parieto-occipital	Mild	Unilateral PRES	Conservative management	Complete	No	No residual deficit
3	Ho and Chan (25)	33/F	Cesarean delivery	140/80	Spinal anesthesia	Headache, altered mental status, disturbed vision, slurred speech, right-sided numbness	2 days	Parieto-occipital	Mild	Cytotoxic edema, diffuse arteries vasospasm	Conservative management	Incomplete	No	No residual deficit
4	Torrillo et al. (28)	32/F	Preeclampsia, cesarean delivery	160/90	Epidural anesthesia	Headache, disturbed vision, buzzing, nausea and vomiting, GTCS	4 days	Superior	Mild	Negative	Conservative management	Complete	No	No residual deficit
5	Hong et al. (26)	29/F	Cesarean delivery	170/100	Spinal anesthesia	Headache, GTCS, left side homonymous hemianopsia	4 days	Superior	Mild	Cytotoxic edema	EBPs, conservative management	Complete	No	No residual deficit
6	Ortiz et al. (9)	33/F	Multiple sclerosis	134/82	Lumbar puncture	Headache, blindness, altered mental status, GTCS	3 days	Parieto-occipital	Mild	Negative	Conservative management	Complete	No	No residual deficit
7	Pradhan et al. (30)	34/F	Renal transplant, prednisolone, daclizumab	Normal	Epidural anesthesia	Headache, GTCS	4 days	Parieto-occipital	Mild	Negative	EBPs, conservative management	Complete	No	No residual deficit
8	Eran and Barak (29)	51/F	Hypertension	144/88	Spinal anesthesia	Altered mental status	1 h	Parieto-occipital	Mild	Cortical and leptomeningeal enhancement	Conservative management	Nearly complete	No	No residual deficit
9	Pugliese et al. (10)	41/F	Cesarean delivery, preeclampsia	Normal	Epidural anesthesia	Headache, mild left motor syndrome, mild right anisocoria, altered mental status, GTCS	7 days	Holohemispheric	Medium	Pachimeningeal enhance	EBPs, conservative management	Nearly complete after 15 days	No	No residual deficit
10	Minai et al. (19)	36/F	Cesarean delivery	Normal	Epidural anesthesia	Neck pain and headache, GTCS, Babinski's sign	3 days	Parieto-occipital	Mild	Negative	Conservative management	ND	ND	No residual deficit
11	Yamada et al. (11)	59/F	Hypertension, ropivacaine	150/80	Epidural anesthesia	Headache, disturbed vision	4 days	Parieto-occipital	Mild	Diffuse arteries vasospasm	Conservative management	Complete	No	No residual deficit
12	Orehek et al. (12)	26/F	Pre-eclampsia	SBP 180	Epidural anesthesia	Headache, GCTS, altered mental status, conjugate left gaze	5 days	Holohemispheric	Medium	IAntracranial hemorrhage	Conservative management	ND	No	Mild left arm dysmetria
13	Sahin et al. (31)	31/F	Cesarean delivery	170/100	Spinal anesthesia	Headache, disturbed vision, GCTS, altered mental status	7 days	Central	Mild	Negative	Conservative management	Incomplete	No	No residual deficit
14	Doherty et al. (32)	19/F	Cesarean delivery	158/91	Epidural anesthesia	Headache, vomiting, photophobia, neck stiffness, disturbed vision, seizure	4 days	Parieto-occipital	Mild	Negative	Conservative management	Complete	No	No residual deficit
15	Grelat et al. (36)	69/F	Chronic hydrocephalus	Normal	Lumbar puncture	Right hemiplegia, altered mental status, deviation to right, disturbed vision, GTCS	12 h	Parieto-occipital	Severe	Negative	Conservative management	Incomplete	Yes	Hemiplegia, difficulties with executive functions
16	Rajan et al. (33)	38/F	Cesarean delivery	Normal	Spinal anesthesia	Headache, GTCS, altered mental status	3 days	Parieto-occipital	Medium	Negative	Conservative management	ND	No	No residual deficit
17	Shah et al. (34)	62/F	Ischemic colitis, hypertension	190/80	Epidural anesthesia	Headache, disturbed vision, blurred discs, status epilepticus	3 days	Parieto-occipital	Severe	Negative	Conservative management	ND	No	Minor visual disturbances and memory problems
18	Hammad et al. (13)	72/M	Hypertension	170/100	Spinal anesthesia	Disturbed vision, altered mental status, GTCS	15 days	Parieto-occipital	Medium	Leptomeningeal enhancement	EBPs, conservative management, external lumbar drain, surgical repair	Complete	No	No residual deficit
19	Feil et al. (16)	19/F	Cesarean delivery	Normal	Epidural anesthesia	Headache, nausea, GTCS, altered mental status, gaze deviation to right	6 days	Central	Medium	Diffuse arteries vasospasm	EPBs, conservative management,	Complete	No	No residual deficit
20	Fok et al. (14)	33/F	Idiopathic intracranial hypertension	142/90	Lumboperitoneal Shunt	Orthostatic headache, GCTS	4 days	Parieto-occipital	Medium	Convexity SAH	Conservative management, removal of lumboperitoneal shunt	Complete	No	No residual deficit
21	Karakis et al. (22)	26/F	Cryptococcal meningitis, AIDS	Normal	Lumboperitoneal Shunt	Seizure, altered mental status	1 day	Parieto-occipital	Medium	Negative	Revision of lumboperitoneal shunt, conservative management,	ND	Yes	No residual deficit
22	Shields et al. (21)	47/F	Hypertension	Normal	Thoracotomy	GTCS, positional headache, altered mental status, disturbed vision	3 days	Parieto-occipital	Severe	Negative	Surgery repair	Minimal residual	No	Mildly blurred vision
23	Santillan et al. (15)	65/F	No	Normal		Headache, altered mental status, left Hoffmann sign	12 days	Parieto-occipital	Medium	Negative	Caffeine, conservative management, EBPs	Complete	No	No residual deficit
24	Sato et al. (20)	79/M	Subarachnoid hemorrhage	Normal	Ventriculo-peritoneal shunt	Headache, altered mental status, left hemiplegia	54 days	Parieto-occipital	Mild	Unilateral PRES	Conservative management	Minimal residual	No	No residual deficit
25	Niwa et al. (37)	72/M	Hypertension, subarachnoid hemorrhage	199/91	Continuous ventricular drainage	Altered mental status, GCTS	6 h	Central	Severe	Negative	Conservative management	Complete	No	No residual deficit
26		68/F	Obstructive hydrocephalus, hypertension	Normal	Cysto- peritoneal shunt placement	Altered mental status, GCTS	1 day	Parieto-occipital	Medium	Negative	Conservative management	Complete	No	No residual deficit
27	Yoon et al. (23)	16/F	Head Trauma, head surgery	SBP 160	Head trauma	GTCS	3 days	Superior	Medium	Negative	Conservative management	Complete	No	No residual deficit
28	Delgado-Lopez et al. (24)	82/F	L4, L5 laminectomy	Hypoten-sion	L4, L5 laminectomy	GTCS, altered mental status	3 days	Parieto-occipital	Medium	Negative	Conservative management	Complete	Yes	No residual deficit
29	Yilmaz et al. (17)	24/F	HELLP syndrome	150/100	Valsalva maneuver	GCTS, altered mental status	ND	Superior	Mild	Negative	Conservative management	Complete	No	No residual deficit
30	Yildiz et al. (35)	23/F	Cesarean section	Normal	Spinal anesthesia	Headache, altered mental status, GTCS	3 days	Parieto-occipital	Mild	Unilateral PRES	Conservative management	ND	No	No residual deficit
31	Present case	30/F	Pregnancy, vaginal delivery	178/96	Epidural anesthesia	Headache, nausea, photophobia, GCTS, diplopia, left abducens nerve palsy, right hemianesthesia, horizontal nystagmus, right tongue paralysis, and right Babinski sign	4 days	Central	Medium	Convexity SAH	Conservative management	Complete	No	No residual deficit

*ND, not described; BP, blood pressure; SBP, systolic blood pressure; CSF, cerebrospinal fluid; GTCS, generalized tonic-clonic seizure; SAH, subarachnoid hemorrhage*.

### Clinical Characteristics

The clinical characteristics of patients with PRES and CSF hypovolemia were listed in Table 2. The median age was 33 years (range: 16–82 years). There was a female predominance (27 females, 87.1%). Thirty patients were associated with one or more known offending factors, most commonly hypertension (*n* = 16), pregnancy (*n* = 14), pre-eclampsia or eclampsia (*n* = 5), subarachnoid hemorrhage (*n* = 2). Five patients had a history of hydrocephalus or intracranial hypertension. Fifteen patients (48.4%) didn't have either a history of hypertension nor an episode of hypertension. The reduction of CSF was resulted from epidural or lumbar puncture (*n* = 21), CSF shunt (*n* = 6), spinal surgery (*n* = 2), head trauma (*n* = 1). Excluding patient 29 who had no exact date of the onset time of PRES (17), the median interval between the procedure leading to CSF reduction and the onset of PRES was 4 days, varying from 2 h to 7 weeks. Headache (71%) was the most common symptom preceding the PRES. Only one patient had a severe elevation of systolic blood pressure more than 200 mmHg. Seizure (83.9%) is the most common neurological symptom in PRES patients with CSF hypovolemia, following by headache (71.0%), altered mental state (64.5%), visual disturbances (41.9%), and hemiparesis (12.9%). Mild edema (51.6%) was most frequent, while the parieto-occipital pattern was most frequent (71.0%). In 80.6% of PRES-CSF hypovolemia patients, follow-up neuroimaging was performed. Of them, complete or nearly complete resolution of edematous lesions was noted in 80.0% of the patients, while 87.1% of the patients had a complete clinical recovery. Three of PRES-CSF hypovolemia patients had a recurrence of PRES after another experience of CSF reduction (22, 24, 36).

**Table 2:** Clinical characteristics and neuroimaging manifestations of patients with PRES and CSF hypovolemia.

**Characteristics**	**Cases, *n* = 31**
Age	33 (26–62)
Gender (Female)	27 (87.1%)
Time to PRES onset (Median, range)	4 days (2 h to 7 weeks)
**Clinical features**
Headache	22 (71.0%)
Seizure	26 (83.9%)
Disturbed vision	13 (41.9%)
Altered mental state	20 (64.5%)
Hemiparesis	4 (12.9%)
Brainstem symptom	3 (9.7%)
Babinski's sign	1 (3.2%)
**Systolic blood pressure (mmHg)**
Normal (<140)	15 (48.4%)
Mild (140–169)	7 (22.6%)
Moderate (170–199)	7 (22.6%)
Severe > 200	1 (3.2%)
**Edema grading**
Mild	16 (51.6%)
Medium	11 (35.5%)
Severe	5 (16.1%)
**Distribution pattern**
Parieto-occipital	22 (71.0%)
Superior	4 (12.9%)
Central	3 (9.7%)
Holohemispheric	2 (6.5%)
Vasculopathy	3 (9.7%)
Complete restitution	20 (80%)^*^
Recurrence	3 (10%)^*^
Favorite outcome	26 (83.9%)

* *The percentages for subcategories are based on the patients who have related data*.

## Discussion

PRES is commonly described as a neuroradiological disease entity characterized by reversible vasogenic edema in the subcortical white matter of bilateral posterior parieto-occipital region with a rapid onset of neurological deficits including seizures, headache, visual disturbances, and altered mental state (2, 4). With the wide application of MR scans, PRES has been much more often recognized in the past decade. The precise pathophysiology underlying PRES is not entirely established. Two contradictory hypotheses are commonly cited (2, 5). The most recognized “Hypertension/hyperperfusion” theory, also called “vasogenic” theory, proposes that severe hypertension, which may overcome the limits of cerebral autoregulation, induces secondary cerebral hyperperfusion leading to an excess of cerebral blood flow, then alterations to the vascular permeability, disruptions to the blood-brain barrier, extravasations of plasma, and subsequent vasogenic edema (2, 5). This concept is primarily supported by the common presence of significant elevation of blood pressure in patients with PRES. Increased perfusion in the vasogenic edema area has been shown in case reports using ASL MRI or CT perfusion (38, 39). Nevertheless, 30–50% of patients with PRES show normal blood pressure or only slightly-to-moderate elevated blood pressure which may not exceed the auto-regulatory limits. The other theory “vasoconstriction/hypoperfusion” theory, or called “endothelial dysfunction” theory, purports that systemic toxicity induces endothelial dysfunction that leads to vascular instability, cerebral vasoconstriction, local hypoperfusion, and subsequent edema (5). This theory is supported by recent vessel imaging and perfusion imaging studies, which have demonstrated diffuse or focal cerebral vasoconstriction, and cerebral hypoperfusion in lesional areas (40). Other proposed theories, such as “cytotoxic” theory, “immunogenic” theory, “neuropeptide” theory, share a similar pathophysiologic mechanism with “vasoconstriction/hypoperfusion” theory (2).

Our case had no stigmata of pre-eclampsia or eclampsia, and the blood pressure maintained normal before and during delivery. She only showed an averaged MAP level of 105 mmHg and a peak mean artery pressure (MAP) level of 123 mmHg after delivery. Did hypertension lead to PRES? Our patient complained postural headache before the changes in blood pressure, and the development of hypertension was following the deterioration of headache. On the other hand, the patient only had a slight elevation of averaged MAP. Even the maximum blood pressure didn't exceed the upper MAP limits of autoregulation. Although puerperium might reduce the threshold of PRES, it is likely that hypertension is not pinpointed as the major cause of PRES. In our review, only 16 patients had hypertension (11–14, 17, 18, 23, 25–29, 31, 32, 34, 37), while only one patient had systolic blood pressure more than 200 mmHg (18). Some patients even experienced hypotension during the development of the disease (11, 24). So, patients with CSF hypovolemia have a different pathophysiological process other than hypertension.

CSF hypovolemia is characterized by orthostatic headaches which almost relive after lying down (6). It was an unequivocal definition of IH characterized by low CSF pressure ( ≤ 60 mmH_2_O). However, nearly half of the IH patients showed normal CSF pressure (8). Even a few patients showed a CSF pressure of more than 200 mmH_2_O (8). So, IH is a clinical syndrome resulting from CSF volume depletion. CSF hypovolemia was proposed to replace the definition of IH (7). The neuroradiological features include pachymeningeal enhancement, brain sagging, subdural fluid collections, pituitary hyperemia, and venous distension sign (41). Although the intracranial pressure was not measured in our case, CSF hypovolemia was well-established on clinical and neuroradiological evidence. Grelat et al. (36) reported a case of chronic hydrocephalus who presented PRES after a depletive lumbar puncture. Interestingly, the patient underwent another episode of PRES following emergency ventriculoperitoneal shunt placement. Similarly, Karakis et al. (22) presented a case of PRES in a patient with IH following lumbo-peritoneal shunt, who experienced PRES 1 week later in the setting of CSF hypovolemia resulting from CSF leakage in the lumbo-peritoneal shunt placement site. Both of them had no other trigger factors. So, it is not surprising that CSF hypovolemia plays a key role in the development of PRES via a different pathophysiology independent of hypertension. In our patient, the ASL imaging provided the evidence of cerebral hyperperfusion in basal ganglion and occipital regions. We speculated that CSF hypovolemia combined with a slight elevation of MAP precipitated PRES by inducing cerebral hyperperfusion. Cerebral perfusion pressure (CPP) is dependent on the relationship between MAP and intracranial pressure (ICP). Depends on the cerebral auto-regulation system, CPP varies from 60 to 80 mmHg. Either increased MAP or decreased ICP will lead to an increase in CPP. When the CPP overwhelms the limits of the cerebral auto-regulation system, cerebral hyperperfusion occurs. Therefore, on the base of CSF hypovolemia, either slightly elevated MAP or normal MAP can lead to cerebral hyperperfusion, endothelial dysfunction, and vasogenic edema (13, 15, 22). On the other hand, the cerebral auto-regulation system ensures a steady ICP in the encephalic space as long as possible. In accordance with the Monro–Kellie doctrine, cerebral blood flow and perfusion in cerebral arteries will firstly increase to maintain normal ICP when CSF leak. If the increased cerebral blood flow and perfusion failed to compensate for the loss of CSF completely, dural sinuses, and veins would engorge for increasing the cerebral blood volume which will lead to capillary and venous hypertension. As a result, fluids extravasated into the interstitial space and vasogenic edema occur. In addition, the brain sagging can result in mechanical traction on the vessels, particularly on the veins of Galen and straight sinus (10, 42). Indeed, the velocity of blood flow in the straight sinus was reported to be declined by an average of 47% in supine patients during and shortly after lumbar punctures (43). Therefore, it impairs the deep venous drainage, induces venous hypertension in the deep venous system, and leads to vasogenic edema dominating in the basal ganglia and occipital regions. To summarize, a combination of arterial hyperperfusion and venous dysfunction may be the pathophysiological link between PRES and CSF hypovolemia.

Some authors hypothesized that reversible cerebral vasoconstriction syndrome (RCVS) secondary to the mechanical stimuli of the sagging of the brain and its affiliations would trigger PRES (11, 16, 25). The pathophysiological mechanism and clinical manifestations of PRES and RCVS partially overlap (16). They share similar triggers, including postpartum, drugs, autoimmune disease, and transplantation. The activation of the adrenergic system is presumed to be key of the development of both diseases (16). In the literature, PRES was observed in nearly 9% of the RCVS patients (44). Vasoconstriction was found in up to 30% of patients with PRES (45). However, cerebral vasoconstriction was found only in three of the patients with PRES and CSF hypovolemia. What draws more attention is that the frequency of RCVS in patients with CSF hypovolemia is particularly low. In a MR-angiography study of a series of 56 patients with IH, only one patient was reported to show segmental stenosis of cerebral arteries (46). There was no evidence of RCVS in our case. As a result, we hypothesize that vasoconstriction/hypoperfusion is not the common etiology of PRES in patients with CSF hypovolemia.

In general, PRES is regarded as a benign disease with favorable outcomes (2, 47). Complete resolution of vasogenic edema and full recovery of neurological deficit were observed in 70–90% of patients. In fact, the poor prognosis was reported in nearly 26–36% cases. Meanwhile, the fatal outcome was documented in 8–17% cases (2). Early identification and rational treatments are crucial to reduce morbidity and mortality. The diagnosis of PRES was usually delayed in patients with CSF hypovolemia until the patients presented with epilepsy and encephalopathy. The most common initial clinical presentations of PRES in patients with CSF hypovolemia is headache which usually misleads to a diagnosis of postdural puncture headache, intracranial hypotension, or pain-related headache. In this regard, the symptom of headache was found to be not of value in the diagnosis of PRES in a retrospective study (48). Only the symptoms of visual disturbances, epilepsy, and encephalopathy are the reasonable predictor of PRES. So, in patients with substantial risk factors of CSF hypovolemia including dural puncture, lumbar puncture, lumboperitoneal shunt, ventriculoperitoneal shunt, and spinal surgery, PRES should be early considered when the clinical manifestations (e.g., epilepsy, visual disturbances, impaired consciousness, focal neurological deficits, resistant headache) could not be entirely explained by CSF hypovolemia, hypertension or other medical condition alone. Multi-spectral MRI sequences, including diffusion-weighted imaging (DWI) imaging, ASL imaging, SWI, and MR angiography, should be performed immediately to establish the diagnosis early, and prevent poor prognosis.

Clinical managements of PRES are based on the elimination of underlying trigger factors and immediate control of epilepsy. Due to the differences in pathogenesis, the treatment strategy for patients with CSF hypovolemia may differ from those with other etiology. Compared with other etiologies, PRES patients with CSF hypovolemia were likely to have a shorter median time from CSF loss to PRES onset, which support a direct link between the CSF hypovolemia and PRES. The time between the procedure that incited CSF loss and the ictus of the PRES syndrome may depend on the baseline ICP and the speed of the reduction of CSF volume or ICP. We found that seven patients experienced PRES within 1 day. Of them, five patients had intracranial hypertension before PRES onset; all of them had a rapid loss of CSF or a rapid reduction of ICP. One patient with chronic hydrocephalus developed PRES 2 h after a rapid CSF loss of 50 ml. The other patient developed PRES 6 h after a 2 h inadvertent overdrainage of 200 ml CSF. These two patients experienced PRES recurrence rapidly after another rapid reduction of CSF volume. On the other hand, a marked increase in blood pressure may contribute to the development of PRES. Patients who experienced a systolic blood pressure more than 179 mmHg had a shorter interval of PRES onset. Base on the evidence from the reviewed reports, we propose the following recommendations: First, a precipitous reduction of CSF volume or ICP should be avoided. A graded reduction of ICP is strongly recommended in patients with intracranial hypertension, especially in patients with extremely high CSF pressures. Second, in patients with CSF hypovolemia, the treatment of CSF hypovolemia should be initiated at the early stage of the disease (49). CSF hypovolemia often recovered spontaneously. Conservative medical management could be processed, including strict supine positioning, ample hydration, analgesia, and non-steroidal drugs. Caffeine and steroids should be avoided due to the risks of RCVS which may induce PRES (9, 15). When the conservative measures failed to bring alleviation of the symptoms or in patients who present moderate and severe CSF hypovolemia, epidural blood patching is recommended as the mainstay of first-line treatment (49, 50). Surgical repair should be considered for patients with clearly identified leak sites and no response to non-surgical treatment and EBPs (50). Third, tight blood pressure control is recommended for patients with CSF hypovolemia due to the increased susceptibility to PRES with a slightly elevated MAP or even normal MAP (13, 15).

## Conclusion

The present case and reviewed literature highlight the pathophysiological link between PRES and CSF hypovolemia. Both arterial hyperperfusion and venous dysfunction may contribute to the development of PRES in patients with CSF hypovolemia. PRES should be early considered in patients with a high risk of CSF hypovolemia when the clinical manifestations can not be explained by CSF hypovolemia or other conditions alone. Precipitous reduction of CSF should be avoided, while appropriate treatments of CSF hypovolemia should be initiated early. The blood pressure should be strictly controlled in patients with CSF hypovolemia to prevent the development of PRES and improve the clinical outcome.

## Data Availability Statement

The raw data supporting the conclusions of this article will be made available by the authors, without undue reservation, to any qualified researcher.

## Ethics Statement

Written informed consent was obtained from the individual(s), and minor(s)' legal guardian/next of kin, for the publication of any potentially identifiable images or data included in this article.

## Author Contributions

FF designed the study. YZ and XW collected clinical data and wrote the manuscript. YC and YL searched the literature and edited the pictures. GZ revised the manuscript. All authors contributed to the manuscript revision and approved the submitted version.

## Conflict of Interest

The authors declare that the research was conducted in the absence of any commercial or financial relationships that could be construed as a potential conflict of interest.
