# Supplementary figures and images for: Cerebrospinal Fluid Hypovolemia and Posterior Reversible Encephalopathy Syndrome
Source: Front Neurol. 2020 Jun 23;11:591. doi: 10.3389/fneur.2020.00591 (PMC7324723; doi:10.3389/fneur.2020.00591)

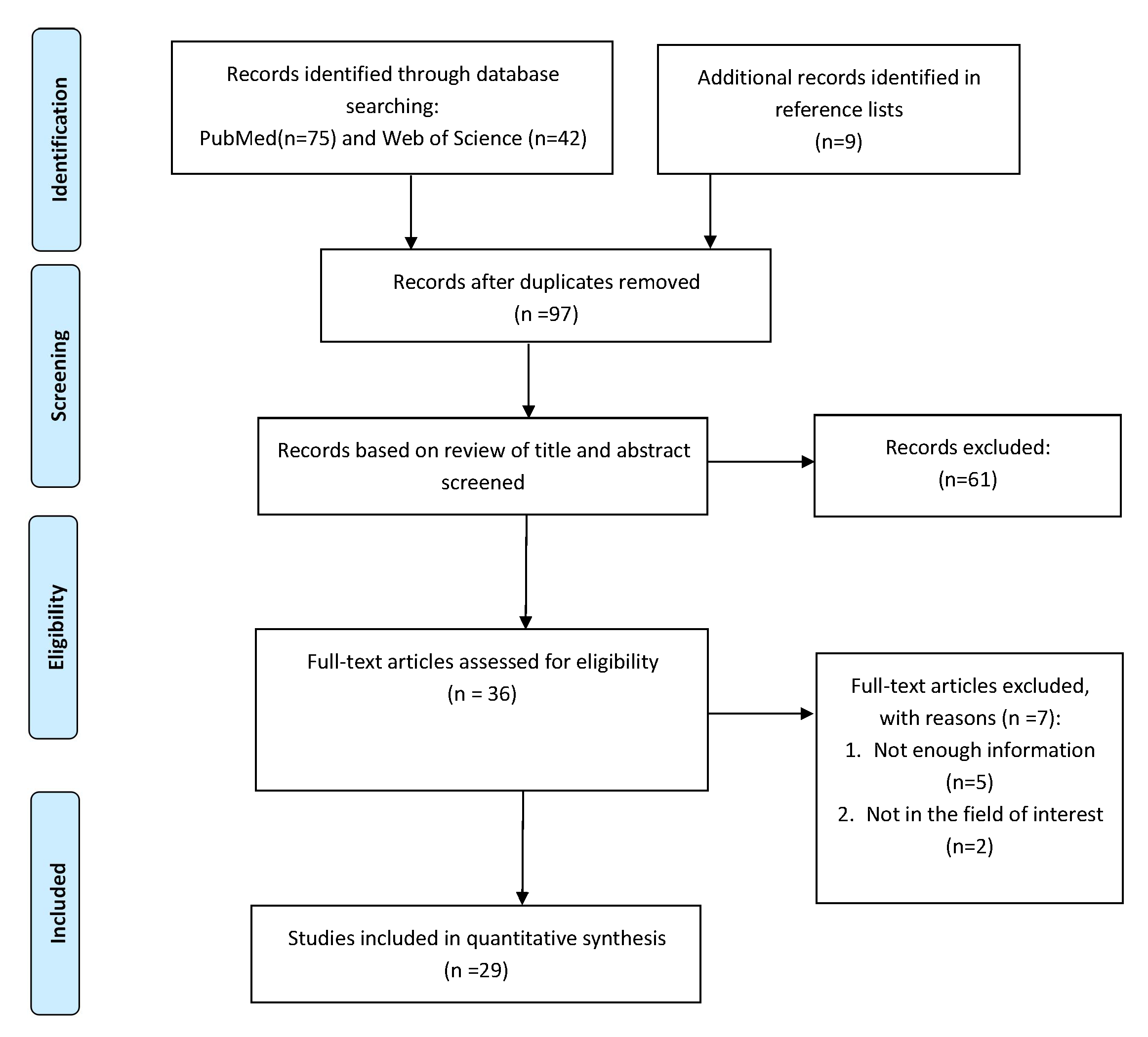

Supplement: Supplementary file 1 [file Image_1.TIF]
